# Parallel Processing Transport Model MT3DMS by Using OpenMP

**DOI:** 10.3390/ijerph15061063

**Published:** 2018-05-24

**Authors:** Linxian Huang, Lichun Wang, Jingli Shao, Xingwei Liu, Qichen Hao, Liting Xing, Lizhi Zheng, Yong Xiao

**Affiliations:** 1 School of Water Conservancy and Environment, University of Jinan, Jinan 250022, China; stu_xinglt@ujn.edu.cn; 2 Engineering Technology Institute for Groundwater Numerical Simulation and Contamination Control, Jinan 250022, China; 3 Department of Geological Sciences, University of Texas, Austin, TX 78705, USA; wanglichun@utexas.edu (L.W.); lizhizheng@utexas.edu (L.Z.); 4 School of Water Resources and Environment, China University of Geosciences (Beijing), Beijing 100083, China; jshao@cugb.edu.cn (J.S.); liuxingwei0912@163.com (X.L.); 5 Institute of Hydrogeology and Environment Geology, CAGS, Shijiazhuang 050000, China; 6 Faculty of Geosciences and Environmental Engineering, Southwest Jiaotong University, Chengdu 610031, China; xiaoyong@cugb.edu.cn

**Keywords:** parallel computing, solute transport modeling, MT3DMS, OpenMP

## Abstract

Solute transport modeling resolves advection, dispersion, and chemical reactions in groundwater systems with its accuracy depending on the resolution of domain at all scales, thus the computational efficiency of a simulator becomes a bottleneck for the wide application of numerical simulations. However, the traditional serial numerical simulators have reached their limits for the prohibitive computational time and memory requirement in solving large-scale problems. These limitations have greatly hindered the wide application of groundwater solute transport modeling. Thus, the development of an efficient method for handling large-scale groundwater solute transport simulation is urgently required. In this study, we developed and assessed a parallelized MT3DMS (Modular Three-Dimensional Multispecies Transport Model) by using OpenMP (Open specifications for Multi-Processing) to accelerate the solute transport simulation process. The parallelization was achieved by adding OpenMP compile directives (i.e., defining various types of parallel regions) into the most time-consuming packages, including the Advection package (ADV), Dispersion package (DSP), and Generalized Conjugate Gradient Solver package (GCG). This allows parallel processing on shared-memory multiprocessors, i.e., both the memory requirement and computing efforts are automatically distributed among all processors. Moreover, we discussed two different parallelization strategies for handling numerical models with either many layers or few layers. The performance of parallelized MT3DMS was assessed by two benchmark numerical models with different model domain sizes via a workstation with two quad-core processors. Results showed that the running time of parallelized MT3DMS can be 4.15 times faster than that using sequential MT3DMS. The effects of using different preconditioners (procedures that transform a given problem into a form that is more suitable for numerical solving methods) in the GCG package were additionally evaluated. The modified strategy for handling numerical models with few layers also achieved satisfactory results with running time two times faster than that via sequential simulation. Thus, the proposed parallelization allows high-resolution groundwater transport simulation with higher efficiency for large-scale or multimillion-cell simulation problems.

## 1. Introduction

Groundwater solute transport modeling is crucial to further our understanding of the transport and fate of pollutants [1,2,3,4,5]. The multispecies transport model MT3DMS, which was originally developed by Chunmiao Zheng of University of Alabama [6], has been the most popular applied and succeeded in simulating groundwater systems over the past several decades [7,8,9]. Nowadays, urgent requirements have been made on modeling groundwater with great refined spatial grids and long temporal scales [10,11]. However, most modeling software is still based on the traditional single-CPU (Central Processing Unit) groundwater simulator and has reached its limits for the prohibitive computational time and memory requirement in handling large groundwater systems. The limitations and challenges of using single-CPU models are particularly due to huge computational ability requirement, large memory capacity requirement, and the restriction on the data channel bandwidth [12,13]. These limitations have greatly hindered the wide application of groundwater solute transport modeling. Therefore, the development of an efficient method for handling large-scale solute transport simulation is urgently required [14]. The solutions to this problem can be roughly classified into two kinds [15]: (1) designing and applying more effective computational technologies, such as new solver (e.g., Algebraic Multigrid Method (AMG), ORTHOMIN, Biconjugate Gradient Stabilized Method (BiCGSTAB), Generalized Minimal Residual Method (GMRES)) and new preconditioner (procedures that transform a given problem into a form that is more suitable for numerical solving methods, e.g., Block-Jacobi, Incomplete LU Factorization (ILU), Incomplete Cholesky Factorization (IC), Domain Decomposition Method (DDM)), to solve the matrix equation resulting from the implicit finite-difference method for MT3DMS; and (2) taking advantage of parallel computing technology. Parallel computing solves large-scale or multimillion-cell simulation problems by allocating and resolving tasks to available multiprocessors simultaneously [16]. Parallel computing has been proved to be a more efficient approach that overcomes the limitations (i.e., constraints on problem size, CPU time, and space resolution) of groundwater simulation [17,18,19,20].

Research on parallel computing for simulating flow process began in the early 1980s. Earlier research mainly focused on petroleum engineering for handling large reservoir problems [13]. Since then, parallel computing technologies in groundwater simulation have developed dramatically. A number of parallel computing techniques have been widely applied to the simulation of groundwater flow and solute transport [15], multiphase flow modeling [2], model calibration [21], groundwater optimization problems [10], uncertainty analysis [21], algorithm development [22], and geothermal engineering [23,24]. Among these parallel computing techniques, MPI (Message-Passing Interface) and OpenMP (Open specifications for Multi-Processing) are considered as the standard parallelization paradigms and have been widely applied [25].

MPI is a message-passing library interface designed for distributed-memory architecture. MPI addresses primarily the message-passing parallel programming problems, in which data is moved from the physical address of one process to that of another process through cooperative operations [26,27]. Many simulators are parallelized via MPI. For example, Zhang, Zhang, Wu and Pruess [2] parallelized TOUGH2 by MPI to handle large-scale modeling of fluid flow in an unsaturated zone at Yucca Mountain. Ashby and Falgout [22] developed a parallelized software of PARFLOW through MPI; PARFLOW is suitable for large-scale problems regarding both saturated and variably saturated flow. Lichtner, et al. [28] presented PFLOTRAN that is parallelized via MPI for modeling multi-phase, multi-component subsurface flow and reactive transport. The above-mentioned parallel software can run on massively parallel computers with hundreds or even thousands of cores. However, parallel computing using MPI is far more complicated. MPI requires users to transform a serial code into a domain decomposed one, where users are responsible to explicitly define how and when data communicates between different processors [25,29,30]. Additionally, the platform setup of MPI is very complicated and time-consuming [31].

In contrast, OpenMP is an application programming interface (API) that supports multi-platform shared memory multiprocessing programming in C, C++, and Fortran [32]. OpenMP API defines a portable, scalable model with a simple and user-friendly interface for developing parallel applications on a variety of platforms, such as desktops and supercomputers [33,34]. Many studies advanced parallel computing through OpenMP. For example, Jin, et al. [35] presented a high-performance hydrobiogeochemistry model HBGC123D (HydroBioGeoChemistry in one, two, and three dimensions) by OpenMP. Dong and Li [15] developed the OpenMP-based PCG (Preconditioned Conjugate-gradient) solver in MODFLOW (Modular Three-dimensional Finite-difference Ground-water Flow Model). McLaughlin [36] implemented OpenMP in the reactive transport model RT3D (Multi-species Reactive Transport Model). Abdelaziz and Le [37] developed a parallelized version of the MT3DMS, namely code-MT3DMSP. The advantage of OpenMP is that it is easy to program and facilitate increment parallelization [33]. Moreover, the platforms for OpenMP are fairly common and cheaply available on the market (such as multicore personal desktop) [34]. This kind of parallel computer is readily available due to the emerging trends of the multicore CPU that combines two or more independent cores into a single package with a single integrated circuit [15]. Due to excellent scalability and convenience, OpenMP has been considered as an ideal parallelization paradigm [38].

Abdelaziz and Le [37] have already developed a parallelized version of MT3DMS code-MT3DMSP, but the simulation time was only reduced from 7.45 min to 3.5 min with a numerical test model that is inefficient. Moreover, MT3DMSP would be less efficient when it refers to numerical models with few layers since not all processors can be used simultaneously. Based on the above analysis, the objective of this study is to parallelize MT3DMS using OpenMP to facilitate groundwater solute transport modeling on shared memory multiprocessors with higher efficiency for numerical models regardless of number of layers. To achieve this goal, first we analyzed the program structure of MT3DMS and identified the code regions that are time consuming. Afterwards, two different parallelizing strategies for handling numerical models with either many layers or few layers were proposed. Then, we added OpenMP directives (defining various types of parallel regions) into these code regions to fully parallelize MT3DMS. We improved the computational efficiency by balancing data communication and load during parallel programming. Finally, the accuracy and efficiency of parallel MT3DMS were demonstrated through two benchmarks for simulating transport problems.

## 2. Methodology

### 2.1. The Governing Equation and the Analysis of Time Consumption for MT3DMS

The governing equation of pollutant transport through a three-dimensional heterogeneous, saturated aquifer is described by the advection-dispersion equation [6,39]:

(1) $$\frac{\partial }{\partial x_{i}}\left(\theta D_{ij}\frac{\partial C^{k}}{\partial x_{j}}\right)-\frac{\partial }{\partial x_{i}}\left(\theta v_{i}C^{k}\right)+q_{S}C_{s}^{k}+\sum R_{n}=\frac{\partial (\theta C^{k})}{\partial t}$$

where *θ* is porosity; *C^k^* is the dissolved concentration of species *k* (ML^−3^); *D_i,j_* is hydrodynamic dispersion coefficient tensor (L^2^T^−1^); *v_i_* is seepage velocity (LT^−1^), *v_i_* = *q_i_/θ*, *q_i_* is specific discharge or Darcy flux, *q_s_* is volumetric flow rate per unit volume of aquifer representing fluid sources (positive) or sinks (negative), T^−1^; *C^k^_S_* is concentration of source or sink flux for species *k*, ML^−3^; *∑Rn* are chemical reaction terms, ML^−3^T^−1^.

The left-hand side of Equation (1) can be expanded into two terms:

(2) $$\frac{\partial \left(\theta C^{k}\right)}{\partial t}=\theta \frac{\partial C^{k}}{\partial t}+C^{k}\frac{\partial \theta }{\partial t}=\theta \frac{\partial C^{k}}{\partial t}+{q_{s}}^{\prime }C^{k}$$

where ${q_{s}}^{\prime }$ = $\frac{\partial \theta }{\partial t}$ is the rate of change in transient groundwater storage (T^−1^).

We consider two basic types of chemical reactions that can be described by:

(3) $$\sum R_{n}=-\rho_{b}\frac{\partial {\bar{C}}^{k}}{\partial t}-\lambda_{1}\theta C^{k}-\lambda_{2}\rho_{b}{\bar{C}}^{k}$$

where $\rho_{b}$ is bulk density of subsurface medium (ML^−1^); ${\bar{C}}^{k}$ is concentration of species *k* sorbed on subsurface solids (MM^−1^); $\lambda_{1}$ is the first-order reaction rate for dissolved phase (T^−1^); $\lambda_{2}$ is the first-order reaction rate for sorbed (solid) phase (T^−1^).

Substituting Equations (2) and (3) into Equation (1) and dropping the species index for simplicity of presentation, this reduces Equation (1) with further rearrangements to:

(4) $$\begin{array}{cc} \theta \frac{\partial C}{\partial t}+\rho_{b}\frac{\partial \bar{C}}{\partial t}= & \frac{\partial }{\partial x_{i}}\left(\theta D_{ij}\frac{\partial C}{\partial x_{j}}\right)-\frac{\partial }{\partial x_{i}}\left(\theta v_{i}C\right)\\ & +q_{S}C_{S}-{q^{\prime }}_{S}C-\lambda_{1}\theta C-\lambda_{2}\rho_{b}\bar{C} \end{array}$$

Equation (4) essentially complies with mass balance, i.e., the change in the mass storage (both dissolved and sorbed phases) at any given time is equal to the difference in the mass inflow and outflow due to dispersion, advection, sink/source, and chemical reactions. For details about deriving Equation (4), readers may refer to the documentation of MT3DMS [6].

By analyzing the governing equation and the program structure of MT3DMS, we find that the most time-consuming code regions are located at Advection package (ADV), Dispersion package (DSP), and Generalized Conjugate Gradient Solver package (GCG). The ADV and DSP Packages solve the concentration change due to advection and dispersion processes, respectively. For a MT3DMS solver using the explicit scheme, both ADV and DSP are compiled to formulate the big coefficient matrix. The GCG solves the matrix equation based on the implicit finite-difference approach via the generalized conjugate gradients methods. Abdelaziz and Le [37] analyzed time consumption of each procedure by running MT3DMS on the Intel^®^ Advisor for a specific numerical model; their results further support our argument that ADV, DSP, and GCG dominantly consumed most of running time (Table 1). In this study, the parallelization of MT3DMS was carried out by adding OpenMP directives into ADV, DSP and GCG packages. Using OpenMP directives, the parallelization can be applied separately to individual subroutines without changing the rest of serial program structure.

### 2.2. Speedup of Parallelization

The performance of parallelized MT3DMS is assessed by the term ”speedup”, which is a standard metric in parallel computing and is defined as the ratio of required time to get work done with only one processor to the time with *N* processors [40]. For example, if *T*(*N*) (parallel MT3DMS) is the time to complete a task on *N* processors and *T*(1) (serial MT3DMS) is the time to finish the task on a single processor, then the speedup *S*(*N*) can be mathematically defined as:

(5) $$S(N)=\frac{T(1)}{T(N)}$$

### 2.3. OpenMP Programming Paradigm

**Figure 1:**
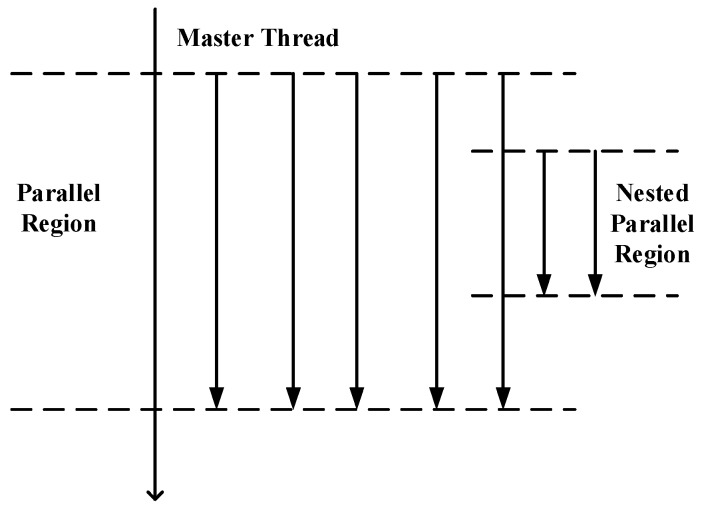
Fork-Join model in OpenMP.

The OpenMP API uses the fork-join model that is executed in parallel (Figure 1) [32]. Multiple threads of execution perform tasks that are either defined implicitly or explicitly by OpenMP directives. At the beginning of program execution, only one thread is active. This thread executes sequentially unless a parallel construct is found. Then this thread creates a team of threads (namely a fork) and itself becomes the master thread. Within the parallel region, the master thread and derived threads work together. Upon completion of the parallel region, these derived threads will quit or hang up, and only the master thread continues, which is called a join. As OpenMP supports the incremental parallelization, it has been widely adopted in scientific computing communities [38]. 

### 2.4. Parallelization of MT3DMS Using OpenMP

#### 2.4.1. Analysis of Parallelization

The advantage of OpenMP is that the parallelization can be done incrementally, that is, the majority of serial code remains unchanged and the users only need to identify and parallelize the most time-consuming parts of serial code, which are usually within loops [9]. This feature is critical for parallelizing the ADV, DSP and GCG packages [10]. Profiling the execution of the sequential ADV, DSP and GCG packages code shows that the block structures with a three-level nested DO-Loop or single DO-Loop takes up most of execution time. The block structures are:

**Figure:**
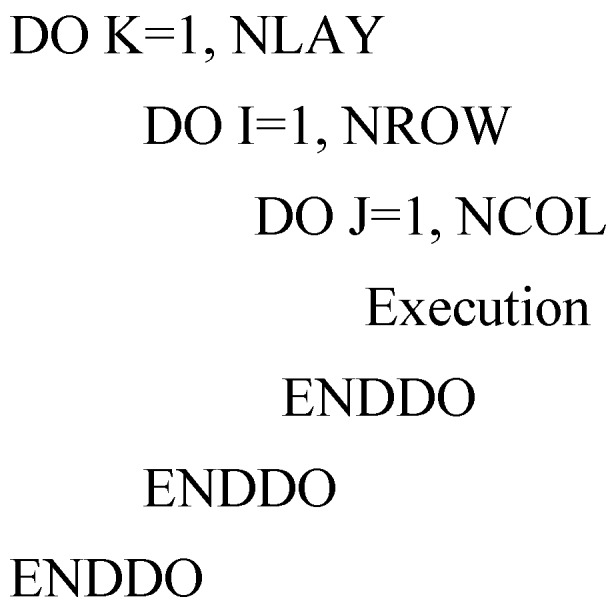

**Figure:**
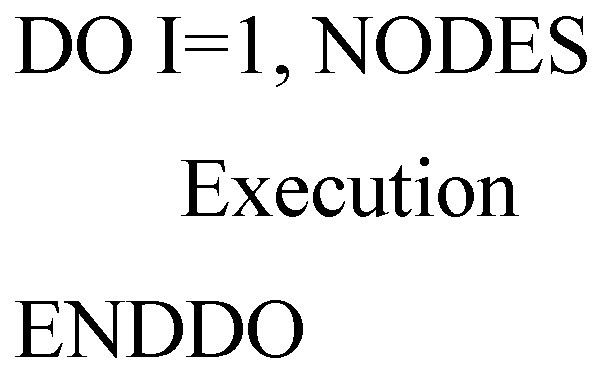

where the variables K, I, and J are the loop counter of each DO-Loop, the variables NLAY, NROW, and NCOL are the total numbers of layer, row, and column in the model, respectively. NODES = NLAY × NROW × NCOL. The parallelization of MT3DMS is achieved by simply adding OpenMP compile detectives into these three-level nested DO-Loop and single DO-loop to significantly speed up execution time and thus improves computational efficiency.

#### 2.4.2. Data Sharing Attribute Clauses

Since OpenMP is a shared memory programming model, most variables are shared to all threads by default [32]. However, private variables are sometimes necessary to avoid race conditions. A race condition occurs if two or more threads access the same variable concurrently and at least one thread induces update [41]. Race condition would result in inaccurate simulation results. Therefore, when parallelized MT3DMS using OpenMP, variables should be defined either shared (variables are shared among all threads) or private (each thread has its own copy of variable).

• Shared clause

When parallelized MT3DMS using OpenMP, certain variables are shared and available to all threads within the scope of a directive-pair. For example, in the above-mentioned three-level nested DO-Loop, NLAY, NROW and NCOL are needed by all threads, therefore these variables can be defined as follows:

!$OMP PARALLEL SHARED(NLAY, NROW, NCOL)

This means all threads have access to the same memory location for reading from/writing to the shared variables. Consequently, declaring a shared variable saves computational memory.

• Private clause

In contrast to the shared variables, some variables should have different values in each thread. This is feasible if every thread has its own copy of variable. The private clause can achieve this by copying the variable that is temporarily existent to each thread. For example, in the above three-level nested DO-Loop, loop counter I, J and K have different values in each thread, therefore these private variables can be defined as follows:

!$OMP PARALLEL PRIVATE(I, J, K)

#### 2.4.3. Reduction Clause

In MT3DMS, there is DO-Loop applied to compute the sum of variables, the block structure can be simply represented as follow:

**Figure:**
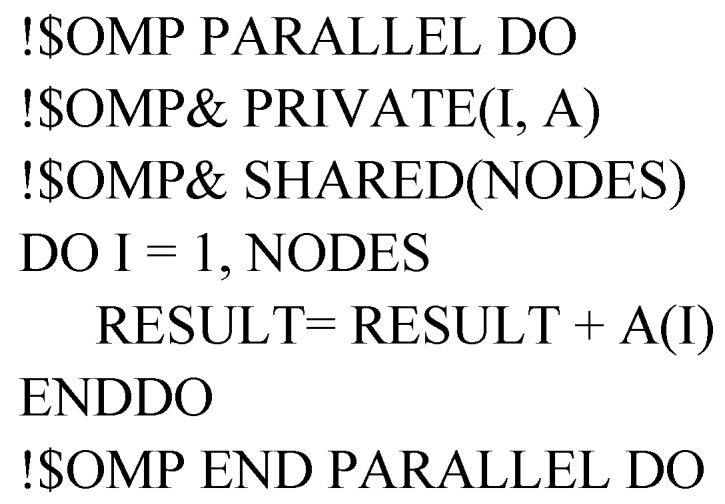

Variable RESULT has a local copy in each thread and the local copies will be summarized (reduced) into a global shared variable. By using the REDUCTION clause, it can help to avoid unpredictable results (see the following block structure). The REDUCTION clause performs a reduction operation on the variable RESULT [32]. At the beginning of reduction, a private copy for RESULT is created and initialized for each thread. At the end of reduction, the local copies will be summarized (reduced) into a global shared variable. Since only one thread at a time is allowed to update RESULT for the shared variable, this ensures the final summation is correct without repeating summing up RESULT.

**Figure:**
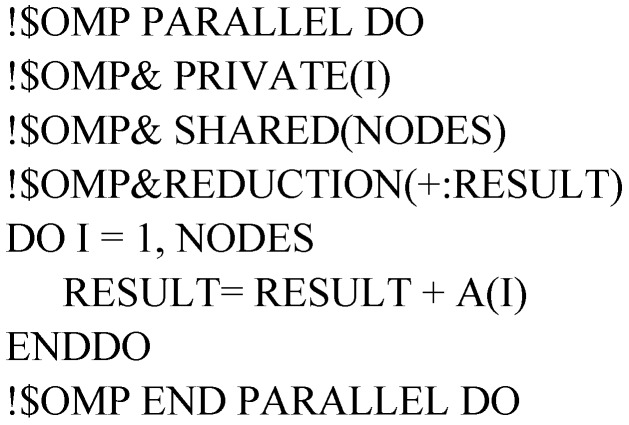


#### 2.4.4. Grain Size

The efficiency of parallelized MT3DMS additionally depends on the grain size. In parallel computing, grain size is a measure of the amount of work (i.e., computation) that is performed by a given task [42]. A large grain size can reduce the amount of available parallelism. Moreover, a small grain size cannot make full use of single processor. Therefore, the balance between grain size and number of processors should be achieved to maximize the computational efficiency. Consequently, there are only two concerned situations regarding the grain size and number of processors, which are either (1) number of layers ≥number of processors or (2) number of layers < number of processors. In this study, we considered these two different situations when we parallelized the three-level nested DO-Loop that included grain size’s effect.

• Case 1: number of layers ≥ number of processors

**Figure 2:**
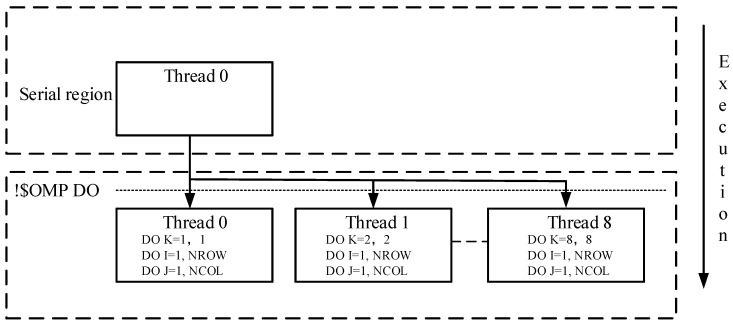
The parallelization of the three-level nested DO-Loop in case of the number of layers > number of processors.

Adding OpenMP compile directives to parallelize the three-level nested DO-Loop can speed up computation on parallel computers. When the number of layers is larger or equal than that of processors (for example, the number of layers and processors are 8 and 2, respectively), the parallelization should be performed on the outer loop because the inner loops are automatically run in parallel if the outer loop is in parallel. For example, if a three-dimension model has 8 layers and the number of thread of multiprocessor computer is 8 as well, parallelizing the K loop should achieve a satisfactory performance since all eight processors can be fully used simultaneously. In addition, the program only incurs the fork/join overhead once. This is shown graphically in Figure 2.

**Figure:**
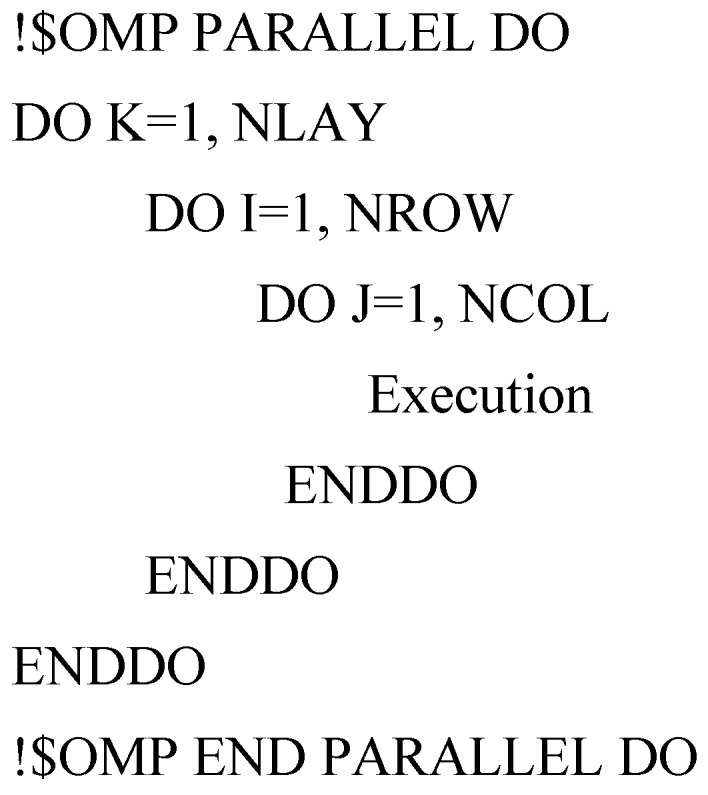


• Case 2: number of layers < number of processors

**Figure 3:**
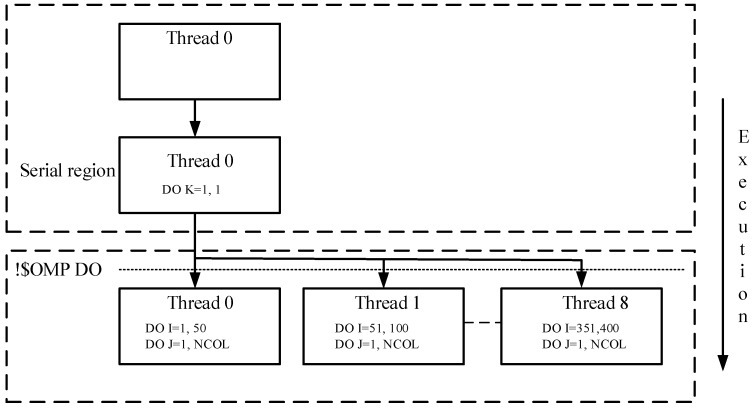
The parallelization of the three-level nested DO-Loop in the case of number of layers < number of processors.

On the contrast, when the model has little number of layers which is less than the number of processors (for example, the number of layers is 2 while the number of processors is 8), the computational performance will be inefficient if we parallelize the outer loop. Specifically, given a two-dimensional model with a single layer but the number of thread of multiprocessor computer is 8; parallelizing the K loop will force the task being processed by only one thread and leave the other seven processors unused; this results in inefficient computation. Note that in such a case, the number of rows or columns maybe very large, so parallelizing I or J loop would be a better option (Figure 3). By parallelizing I or J loop, the K loop will be performed in a serial mode using only one processor, but the I or J loop will be processed in parallel with all eight processors.

**Figure:**
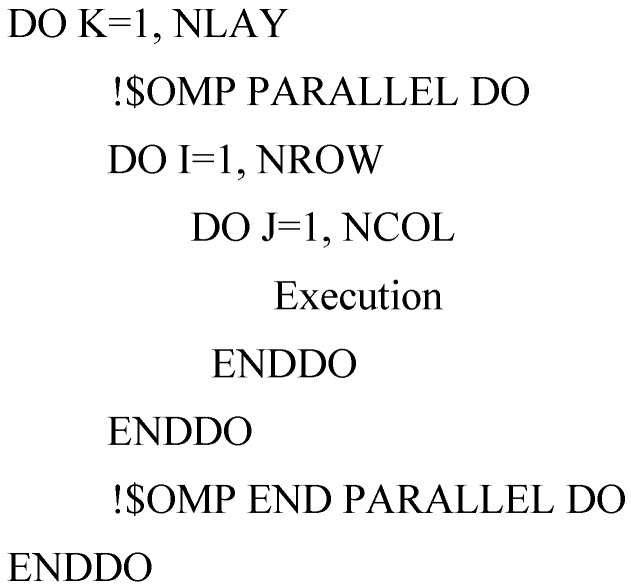


#### 2.4.5. Other Issues Need Be Addressed for Parallelization

There are relevant issues when parallelizing the blocks with OpenMP.

- The time-stepping loops and iteration loops are not parallelizable because the results are interconnected between two successive time steps and iterations.
- The code enclosed in a parallel region must be a structured block of code. That is, it is not allowed to jump in or out of a given parallel region, for example, by using a GOTO command.

## 3. Performance Results

The performance and robustness of the parallelized version of MT3DMS was assessed by two benchmark numerical models as discussed below. All tests were performed on a Windows workstation equipped with two quad-core 2.4 GHz Intel Xeon-E5620 processors. The metric ”speedup” was used to measure the performance of parallel computing. The MT3DMS 5.3 serial program was also run for the sake of comparison. All numerical codes were compiled by Intel^®^ Visual Fortran Composer XE 2013 on Windows 8.

### 3.1. Benchmark Test 1

A numerical model P07 consisting of 21 columns, 15 rows, and 8 layers was used to solve the three-dimensional transport problem; this model was taken and modified from the manual document of MT3DMS. More detailed information of this model can be found in the document of MT3DMS [6]. Eight processors were used to assess the performance of parallel computing.

#### 3.1.1. Comparison between Parallel MT3DMS and Serial MT3DMS

**Figure 4:**
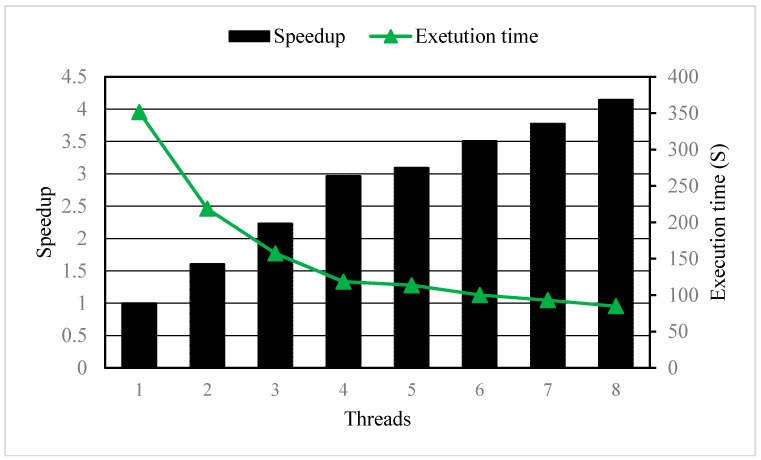
The Speedup and execution time with different number of threads.

**Figure 5:**
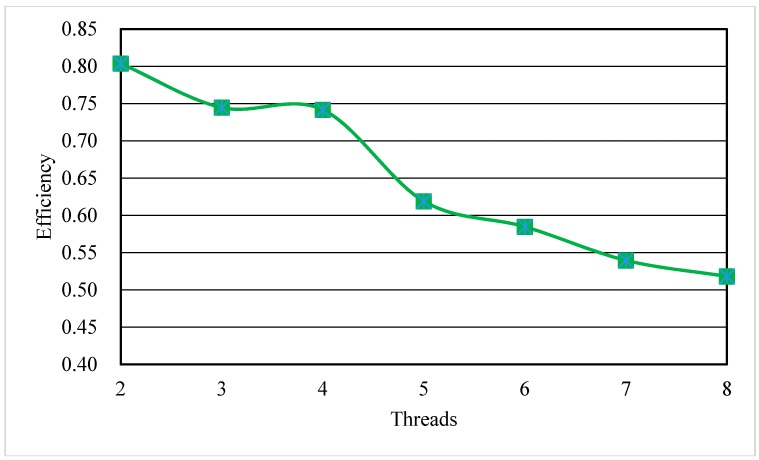
The efficiency with different number of threads.

These numerical models were simulated separately by the parallelized MT3DMS and serial MT3DMS. The execution times and speedup of the parallelized MT3DMS with different number of threads are shown in Figure 4. The parallelized MT3DMS can significantly shorten the execution time and speed up the computation with the running time 4.15 times faster when using 8 processors than that when using single processor. As expected, the speedup increased and the execution time declined with increasing number of threads. However, we observed that the efficiency (=speedup/number of threads) was declined when the thread number increased from 5 to 8 as shown in Figure 5. The reduction of efficiency were mainly due to the system overhead of synchronization between threads, overload, data race problems, creation threads, and hang up threads.

#### 3.1.2. Comparison between Three GCG Preconditioning Options

The Generalized Conjugate Gradient Solver package (GCG) has three preconditioning options, Jacobi, Symmetric Successive Over Relaxation (SSOR), and the Modified Incomplete Cholesky (MIC) for solving the matrix equation. All three preconditioning options have been parallelized using OpenMP to some degree. The performance of parallel MT3DMS with these three preconditioning options were furthered assessed as following.

**Figure 6:**
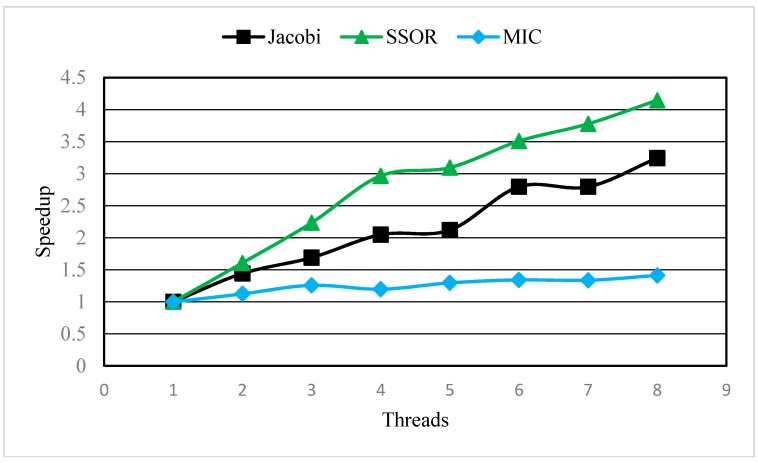
The dependence of speedup on the number of threads for different preconditioning options.

**Figure 7:**
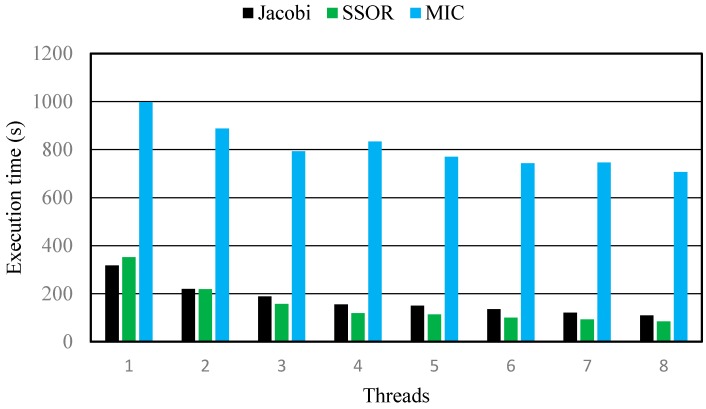
Execution time vs. number of threads for different preconditioning options.

The SSOR preconditioner and Jacobi preconditioner perform very well while the MIC preconditioner performs poorly in terms of speeding up computation (Figure 6) and shortening the running time (Figure 7). When the number of threads was 1, the execution time of the MIC preconditioner was about 1000 s which was nearly 3 times longer than that of the SSOR and Jacobi preconditioners. The main reason is that the MIC preconditioner usually takes more iterations to converge than the Jacobi or SSOR and demands much more memory [6]. Both the SSOR and Jacobi preconditioners can significantly reduce the execution time with increasing number of threads, however the SSOR preconditioner are faster than the Jacobi preconditioner when the number of threads >2 (Figure 7); the maximum speedup of the SSOR and Jacobi preconditioners are 4.15 times and 3.24 times, respectively, when the number of threads was 8 (Figure 6). These results indicate that the parallelization of the SSOR and Jacobi preconditioners can pronouncedly improve the computational efficiency. On the contrary, the MIC preconditioner produces only a marginal difference in the execution time with increasing threads, in which the maximum speedup was only 1.41; this suggests the parallelization of MIC preconditioner is ineffective.

In short, both parallel SSOR and Jacobi preconditioners are effective for improving computational efficiency; therefore, they are a good choice for simulating groundwater solute transport problems with massive grids.

### 3.2. Benchmark Test 2

In order to evaluate the influence of grain size, an exploratory numerical model HSSTEST consisting of 46 columns, 31 rows, and only 1 layer was taken and modified from the manual document of MT3DMS [6]. Based on model HSSTEST, we further show the effects of different strategies in parallelizing MT3DMS when number of layers is less than the number of processors.

#### 3.2.1. Parallelizing the K Loop

**Figure 8:**
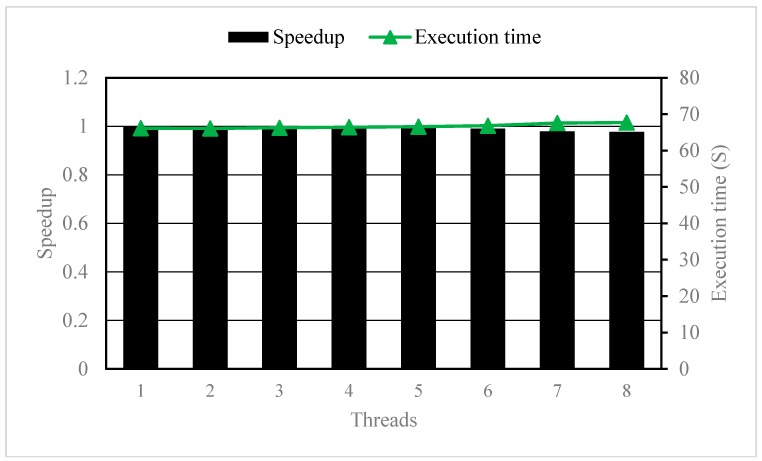
The Speedup and execution time with different number of threads when parallelizing the K loop.

We added OpenMP compile directives to parallelize the K loop (i.e., the layer loop) of the three-level nested DO-Loop. The execution time and speedup were shown in Figure 8. Apparently, the execution time and speedup only had a marginal difference with increasing thread numbers. This is because the number of layers (=1) was far less than the number of processors, so only one processor was performed during the entire simulation, which was remarkably inefficient since the other seven processors were not used.

#### 3.2.2. Parallelizing the I Loop

**Figure 9:**
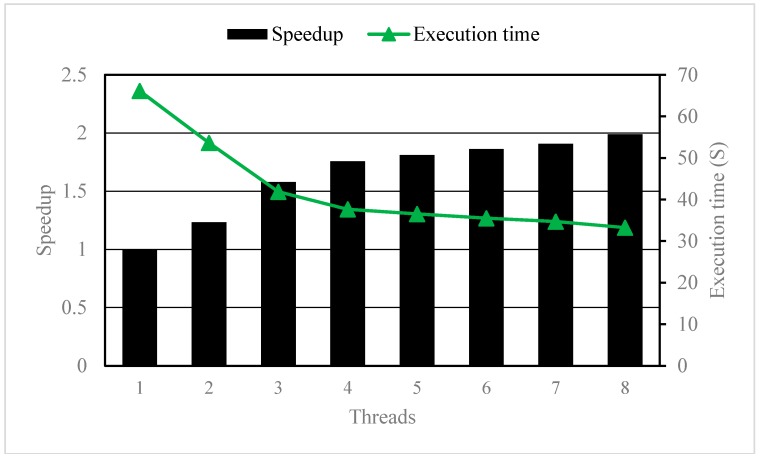
The Speedup and execution time with different number of threads when parallelizing the I loop.

We further modified the OpenMP compile directives to the I loop (i.e., the row loop) of the three-level nested DO-Loop. As a result, the K loop will run in serial with only one single processor and the I and J loop (i.e., the column loop) will run in parallel with all eight processors. The execution time and speedup were shown in Figure 9. For example, the maximum speedup was about 2 when the thread number was 8. Furthermore, the speedup increased with thread number. The results indicated that more satisfactory performance can be achieved by parallelizing the I loop than that by parallelizing the K loop. Note that in real-life cases for groundwater transport simulations, the number of layers are usually very small while the number of rows or columns are usually very large. Therefore, parallelizing the I loop unarguably outperforms that of parallelizing the K loop since all processors can be fully used.

## 4. Conclusions

The traditional serial solute transport numerical simulators have challenges in solving large-scale problems due to the huge computational ability requirement, large memory capacity requirement, and the restriction on the data channel bandwidth. To address this challenge, a parallelized version of MT3DMS is developed to speed up the groundwater solute transport simulation with fine mesh discretization and long time periods by taking advantage of multi-core shared memory computers. The parallelization of MT3DMS is accomplished by adding OpenMP compile directives into three-level nested DO-Loop and a single DO-Loop of MT3DMS. The performance of parallelized MT3DMS was assessed by two benchmark numerical models. The results showed that the parallelized MT3DMS can effectively shorten the execution time and improved the computational efficiency. In the first benchmark, the maximum speedup of 4.15 times could be achieved for an 8-layer numerical model when thread number was 8; this demonstrates a significant improvement for the parallelized MT3DMS. We additionally compared the three preconditioning options of Generalized Conjugate Gradient Solver package. Results indicated both the Symmetric Successive Over Relaxation and Jacobi preconditioners performed very well while the Modified Incomplete Cholesky preconditioner performed poorly in terms of computational efficiency. In order to handle the situation when the number of layers of numerical model is less than the number of processors, we further modified the parallelization strategy by parallelizing the row loop instead of the layer loop. The performance of this modification was assessed in the benchmark 2 for a one-layer numerical model. In this case, we found that parallelizing the row loop is more efficient than parallelizing the layer loop.

Overall, this study developed a novel parallelized version of MT3DMS to resolve problems of massive groundwater solute transport simulation. This study is not the end of parallel computing; however, our study serves as the first step that shows the advantage of parallelizing MT3DMS using OpenMP. Further developments are also necessary to improve computational efficiency. Currently, not all the packages have been parallelized and more packages will be parallelized in future. In order to further improve the efficiency, the input and output formats of hydrogeologic data have to be in parallel in future as well. Moreover, the current application of proposed parallelized MT3DMS is restricted in a shared-memory architecture, so the hybrid OpenMP/MPI approach would be applied to ensure it can run on either shared-memory architecture or on distributed-memory architecture to take advantage of more advanced parallel computers. More case studies are needed to additionally demonstrate the applicability of the parallelized model.

## Figures and Tables

**Table 1:** Time consumption for different packages of MT3DMS.

Package	Fraction of Time Consumption
ADV	63%
DSP	20%
GCG	13%
Others	4%
